# The PSY Peptide Family—Expression, Modification and Physiological Implications

**DOI:** 10.3390/genes12020218

**Published:** 2021-02-02

**Authors:** Amalie Scheel Tost, Astrid Kristensen, Lene Irene Olsen, Kristian Buhl Axelsen, Anja Thoe Fuglsang

**Affiliations:** 1 Department of Plant and Environmental Sciences, University of Copenhagen, Thorvaldsensvej 40, DK-1871 Frederiksberg, Denmark; plv212@alumni.ku.dk (A.S.T.); astridpariserlinse@gmail.com (A.K.); fjb225@alumni.ku.dk (L.I.O.); or kax@plen.ku.dk (K.B.A.); 2 SIB Swiss Institute of Bioinformatics, CMU, 1 Rue Michel Servet, CH-1211 Geneve, Switzerland

**Keywords:** **Keywords**: signaling peptide, sulfated tyrosine, expression analysis, cell elongation, root growth

## Abstract

Small post-translationally modified peptides are gaining increasing attention as important signaling molecules in plant development. In the family of plant peptides containing tyrosine sulfation (PSYs), only PSY1 has been characterized at the mature level as an 18-amino-acid peptide, carrying one sulfated tyrosine, and involved in cell elongation. This review presents seven additional homologs in *Arabidopsis* all sharing high conservation in the active peptide domain, and it shows that PSY peptides are found in all higher plants and mosses. It is proposed that all eight PSY homologs are post-translationally modified to carry a sulfated tyrosine and that subtilisin-like subtilases (SBTs) are involved in the processing of PSY propeptides. The PSY peptides show differential expression patterns indicating that they serve several distinct functions in plant development. PSY peptides seem to be at least partly regulated at the transcriptional level, as their expression is greatly influenced by developmental factors. Finally, a model including a receptor in addition to PSY1R is proposed.

## 1. Introduction

The highly complex network of cell-to-cell communication is crucial for the regulatory mechanisms involved in plant development and stress responses [1]. During the last couple of decades, the scientific interest in secreted peptides as important signaling molecules has emerged and increased. Signaling peptides are involved in many aspects of plant regulation including developmental processes and responses to biotic and abiotic stress factors [2,3,4]. Concurrent with the increased focus on signaling peptides, the number of characterized types has increased considerably and now exceeds the number of classical plant hormones [4]. However, the physiological roles and precise functions of the many signaling peptides still remain to be elucidated.

The plant peptides containing sulfated tyrosine (PSYs) were initially identified by Amano et al. 2007 [5]. Nevertheless, the PSY family has still not been characterized in detail. Although the physiological role of PSY1 has been partly established, the role and importance of the whole PSY peptide family in plant cell communication has not been fully elucidated. This review offers an overview of published material of the PSY family in addition to a meta-analysis based on material found in the expression databases.

## 2. Small Tyrosine-Sulfated Peptides

### 2.1. Post-Translational Modifications

Signaling peptides are derived from prepropeptides, which are precursor peptide sequences of approximately 70–200 amino acids. These prepropeptides are often encoded by families of homologous genes, and consequently, the outcome might be functionally overlapping isoforms of secreted peptides [4,6]. Prepropeptides contain N-terminal signal peptides that direct them into the secretory pathway, where cleavage of the secretion signal and further modifications are needed to get functionally active, mature peptides [3]. Secreted signaling peptides are categorized into two classes: small post-translationally modified peptides and cysteine-rich peptides. Plant peptides containing sulfated tyrosine (PSYs) belong to the former and are the focus of this review. 

Post-translationally modified peptides are modified and processed in the secretory pathway before secretion, typically modifications can be tyrosine sulfation, proline hydroxylation and hydroxyproline arabinosylation [3,7]. Phytosulfokine (PSK) was the first tyrosine-sulfated peptide to be discovered in plants as a five-amino-acid peptide carrying two tyrosine sulfations essential for its activity [8,9]. Four types of tyrosine sulfated peptides have now been identified in plants; PSKs, root growth meristem factors (RGFs), casparian strip integrity factors (CIF), and plant peptides containing sulfated tyrosine (PSYs) [4,10]. 

**Figure 1:**
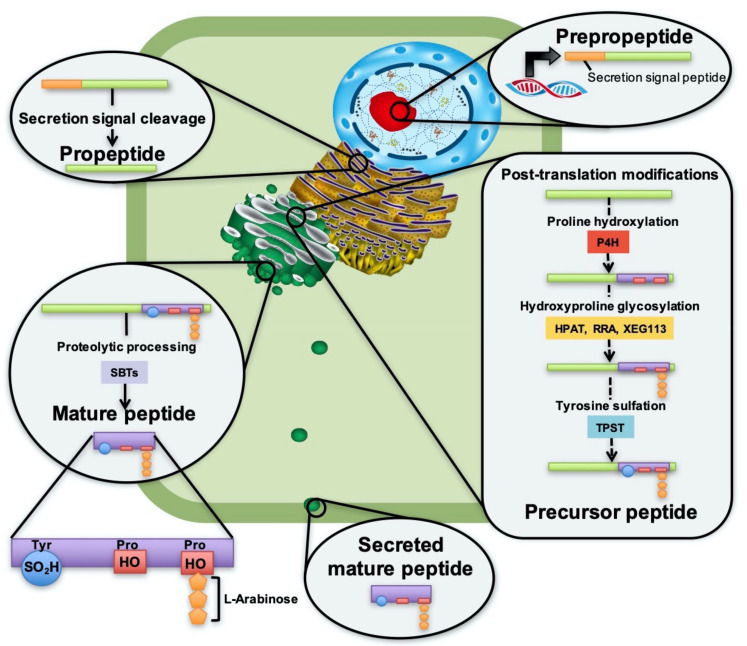
Proposed PSY synthesis pathway and suggested enzymes involved in PSY processing. PSY genes are expressed in the nucleus as prepropeptides carrying a secretion signal peptide. The secretion signal is cleaved upon arrival in the endoplasmic reticulum (ER), revealing the propeptide. The propeptide is received in the cis-Golgi where it undergoes post-translational modifications to become a precursor peptide. Prolyl-4-hydroxylases (P4H) catalyze hydroxylation of proline. Hydroxyproline O-Arabinosyltransferases (HPAT) catalyze the attachment of a L-Arabinose unit to one of the two hydroxyl groups. Reduced Residual Arabinose (RRA) and Xyloglucanase113 (XEG113) are proposed to attach the second and third arabinose unit, respectively. Tyrosylprotein sulfotransferase (TPST) catalyzes the sulfation of the tyrosine residue. Before reaching the Trans-Golgi network, the precursor peptide is proteolytically processed to release the mature peptide. Subtilases (SBTs) and in particular SBT3.8 and SBT6.1 are suggested to be involved in PSY processing. The mature peptide is transported from the trans-Golgi to the cell membrane in vesicles, where it fuses with the membrane to secrete the mature peptide to the apoplast.

PSY1 was isolated from *Arabidopsis* cell culture medium and identified as an 18-amino-acid residue carrying three post-translational modifications in its active domain; one sulfated tyrosine and two hydroxylated proline residues, one of which is also glycosylated with three L-arabinose units [5]. These modifications are all catalyzed by processing enzymes in the secretory pathway. Figure 1 presents the proposed synthetic pathway of PSY peptides from expression in the nucleus to secretion into the apoplast, and suggests enzymes involved in processing to reveal the mature, active peptide.

Tyrosine sulfation is catalyzed by tyrosylprotein sulfotransferase (TPST), which is encoded by only a single gene in *Arabidopsis* [11,12] (Figure 1). Sulfation catalyzed by TPST is highly site specific and replacement of acidic amino acid in the −1 or +1 position to tyrosine decreases TPST activity [13]. Tyrosine sulfation has proven to be essential for PSY maturation, as *tpst* mutants are characterized by serious developmental defects, a phenotype which was rescued by treatment with PSY1, PSK, and RGF1 [14]. This may be explained by sulfate providing stability to the peptide when secreted into the protoplast and also increasing binding affinities to its receptor [15,16].

**Figure 2:**
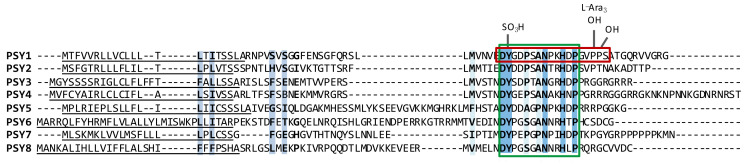
Alignment of the PSY1 precursor peptide and its seven homologs in *Arabidopsis*. Letters in bold and shades of blue indicate conservation between amino acids with weakly similar properties, amino acids with strongly similar properties, and complete conservation, respectively. Green box highlights the conserved PSY domain and red box emphasizes the mature, active PSY1 peptide with indicated modifications. Predicted secretion signal peptide is underlined. PSY5 is found in four splice variants, of which the shortest is presented in the figure.

Proline hydroxylation, another type of modification, is catalyzed by prolyl-4-hydroxylases (P4H), which are type II membrane proteins with an N-terminal transmembrane domain, localized in the ER and Golgi (Figure 1). Thirteen homologs have been found in *Arabidopsis*, but which of these are involved in PSY maturation [16,17] has still not been identified. So far no consensus sequence motif has been found for proline hydroxylation in plants [18], although a motif has been suggested by Shimizu et al. 2005 [19] and examined in Vlad et al. 2007 [20]. In *Arabidopsis*, substrate specificity has been shown for P4H2 [21] and P4H5 [22]. There are no obvious shared consensus sequences between the hydroxylated prolines of PSY1 and the PSY2-8 isoforms (Figure 2), and it remains to be determined experimentally whether these isoforms are hydroxylated and possibly glycosylated in vivo.

Lastly, one of the two hydroxylated prolines is glycosylated at the hydroxyl group with three L-arabinose units (L-Ara_3_) (Figure 1). Hydroxylation alone is often not important for protein conformation and activity of the peptide, but instead serves as a required bridge for the arabinosylation, which is critical for maintaining proper peptide structure and thus activity [3]. The first arabinose is added by hydroxyproline *O*-arabinosyltransferases (HPAT) of which three homologs have been identified in *Arabidopsis* [23]. HPAT1, HPAT2, and HPAT3 have been found to catalyze this reaction in vitro of a synthetic peptide carrying the same arabinosylation site as PSY1 [23]. It remains to be elucidated which homolog(s) are involved in PSY1 O-glycosylation. Attachment of the second and third L-arabinose unit is proposed to be catalyzed by Reduced Residual Arabinose 1-3 (RRA1-3) and Xyloglucanase113 (XEG113), respectively, or other arabinosylltransferases of the GT77 family, as is the case for extensins carrying O-Arabinosylation of hydroxyprolines identical to that of PSY1 [17,24,25].

### 2.2. Peptide Processing 

In addition to post-translational modifications, PSYs are characterized by undergoing proteolytic processing to liberate the mature, active peptide (Figure 1). Recent studies have demonstrated processing as a checkpoint during peptide maturation, and it is now believed that proteolytic processing of the prepropeptides involves several steps and that both initial endoproteolysis and subsequent fine-trimming exoproteolysis take place [4]. Precursor processing enzymes in three different classes of proteases have been identified in *Arabidopsis*; cysteine peptidases, zinc-dependent metallo peptidases, and serine peptidases [26]. Among these, subtilases (SBTs) of the serine family have been shown to be involved in processing of multiple post-translationally modified peptides, including the tyrosine sulfated PSKs and RGFs [26,27,28]. 

### 2.3. Physiological Implications of PSYs

The biosynthesis of posttranslational modifications is a very energy-consuming process and, in particular, tyrosine-sulfation is very costly for the plant [4,5]. Small peptides carrying a sulfated tyrosine are thus alleged to serve an important function in plant development. 

PSY1 is the only investigated peptide of the PSY family to date, and it is expressed in various tissues throughout the plant [4,29]. The PSY1 signaling pathway includes the leucine-rich repeat receptor-like kinase, PSY1 receptor (PSY1R) [5], which upon ligand activation phosphorylates and thus activates the plasma membrane (PM) H^+^-ATPase AHA2 [30]. This results in increased H^+^ efflux as measured by non-invasive ion flux measurements (MIFE). In the *psy1r* receptor mutants this response is significantly reduced, but not completely lacking, which indicates the presence of yet another receptor or co-receptor for PSY peptides. The proton transport generated by PM H^+^-ATPases creates the proton-motive force, that provides the energy for most other secondary active transporters in the PM. PM H^+^-ATPases are involved in cell elongation and stomata regulation, and help the plant adapt to biotic and abiotic stresses [31,32], thus suggesting a regulatory role for PSY1 in cell expansion and proliferation. PSY1-induced cell elongation has been demonstrated in the roots [5,33] and hypocotyls [30]. Moreover, PSY1 plays a role in regulation of plant immunity [34]. Here, PSY1 signaling is induced by pathogen infection and involved in downregulation of the salicylate-related responses. This prevents an extended activation of this particular pathway which would otherwise leave the plant more vulnerable to other pathogens [34].

In a study by Pruitt et al. (2017) [33] it was found that the biotrophic pathogen *Xanthomonas oryzae pv. oryzae* (*Xoo*) produces a sulfated peptide, RaxX, with similarity to peptides in the PSY family [33]. The conserved PSY domain is highly conserved in RaxX, harboring both the DY, N, H, and P motifs conserved in all PSY peptides (Figure 2). RaxX serves as a molecular mimic of PSY peptides to facilitate *Xoo* infection. Interestingly, the *psy1r-1* mutant still responded to the RaxX peptides, indicating that PSY1R was not the receptor for RaxX. It was found that the receptor XA21 recognized RaxX but not AtPSY1, indicating that XA21 has evolved the ability to recognize and respond specifically to the microbial form of the peptide and not to the plant version [35]. This was demonstrated by binding studies, in which RaxX peptides bound to the ectodomain of XA21, in contrast to PSY1 [35]. The authors hypothesized that PSY1 and RaxX targeted a common cognate plant receptor. The authors did not show whether RaxX stimulated H^+^ extrusion like PSY1 does. 

## 3. Materials and Methods 

### 3.1. Bioinformatics

Potential PSY peptide homologs were identified using the NCBI protein BLAST server (www.blast.ncbi.nlm.nih.gov/Blast.cgi; [36]). The full precursor sequence of PSY1 was used as input query. To be considered a putative homolog, the following criteria must be met: peptides must match input query with expect value ≤20, PSY-like motif must begin with Asp-Tyr, and the full-length protein must be between 60 and 200 amino acids with PSY-like motif in the second half. The accession numbers of all hits were used to retrieve FASTA sequences at the UniProtKB server (www.uniprot.org; [37]). Multiple alignment was made using MUSCLE (www.ebi.ac.uk/Tools/msa/muscle/; [38]) and pairwise global alignments were performed using the Needleman-Wunsch algorithm at www.ebi.ac.uk/emboss/align. For pairwise alignment the BLOSUM62 matrix was used with gap opening penalties of 10 and gap extending penalties of 0.5. Secretion signals and cleavage sites were predicted using the SignalP 5.0 server with default setting (http://www.cbs.dtu.dk/services/SignalP; [39]). 

Based on the results from the BLAST searches, the motif (DYXXXX[AP]NXXHXP) was generated and used to scan the UniProtKB database with the ScanProsite tool at https://prosite.expasy.org/scanprosite/ [40] to possibly find PSY homologs that might have been missed by the BLAST searches.

Alignment of PSY homologs from plants and mosses was based on translated complementary DNA sequences and was made using MUSCLE [38]. The following amino acid sequences were used in the alignment: *Arabidopsis thaliana* AtPSY1 (Q941C7), AtPSY2 (Q8LE92), AtPSY3 (Q8S8P7), AtPSY4 (B3H674), AtPSY5 (Q1G3G7), AtPSY6 (A0MDK8), AtPSY7 (Q3E7N2) and AtPSY8 (Q9SN87); *Amborella trichopoda* AmtPSY (W1PCD7; genome sequence); rice (*Oryza sativa)* OsPSY1 (A2ZX08), OsPSY2 (Q5TKN9), OsPSY3 (Q5NBP8), OsPSY4 (Q9LDG6), OsPSY5 (Q5N756), OsPSY6 (Q75KZ0) and OsPSY7 (B9FKV0); maize (*Zea mays)* ZmPSY1 (B6TP25), ZmPSY2 (B6U638), ZmPSY3 (B6SX29), ZmPSY4 (B6UG95), ZmPSY5 (B6TWC1), ZmPSY6 (B6SJ20), ZmPSY7 (B6SK20) and ZmPSY8 (B6T2H1); soybean (*Glycine max*) GmPSY1 (C6T043), GmPSY2 (C6SWX4) and GmPSY3 (C6T324); chickpea (*Cicer arietinum*) CaPSY1 (Q8GTD8); *Lotus japonicus* LjPSY1 (I3SPZ1); *Populus trichocarpa* PtPSY1 (A9P872); *Medicago truncatula* MtPSY1 (A2Q4B1) and *Physcomitrella patens* PpPSY (A0A2K1K614).

### 3.2. Genevestigator Expression Profiles

PSY expression patterns in different plant anatomical parts and at different developmental stages were obtained from Genevestigator [41]. The mRNAseq data selection was used as all seven PSYs are represented in this dataset. On the standard ATH1 microarray chip only PSY1-3 are represented. Only experiments with *Arabidopsis* of wildtype genetic background were included in the analysis. For tissue- and development-specific transcript data, only samples not receiving any experimental treatments were included. All experiments with less than three samples were excluded.

### 3.3. Plant Material and Growth Conditions

Wildtype *Arabidopsis thaliana* Col-0 seeds were surface sterilized and sown on half strength MS media (½ MS, 1% sucrose, 1% agar, pH 5.7) and stratified in darkness at 4 °C for 2 days. Seeds were grown in a climate chamber maintained at 22 °C and an 8 h-light/16 h-dark cycle. Plant material was harvested at indicated time points, frozen in liquid nitrogen, and kept at −80 °C until used for RNA extraction.

### 3.4. Quantitative Gene Expression Analysis

Total RNA was extracted using the Plant RNA Kit (Omega) and subsequently treated with DNA-free (Ambion). First strand cDNA was produced using iScript (Biorad). qPCR was performed with Brilliant II SYBR Green QPCR Master Mix (Agilent Technologies) on an Mx3000P cycler from Stratagene. Gene-specific transcripts were normalized to the level of actin-2 transcript. Primer sequences used for qPCR are listed in Table 1. Data analysis was performed using the REST program based on comparative C_t_ method (∆∆C_t_) [42]. All qPCR data represent three biological replicates each with two technical replicates. 

## 4. Results and Discussion

### 4.1. PSY1 Has Seven Homologs in Arabidopsis

PSY1 has previously been reported to have two homologs in *Arabidopsis* [5], now named PSY2 and PSY3. A search for additional homologs using the active PSY1 peptide sequence as query and scanning UniProtKB with the motif (DYXXXX[AP]NXXHXP) based on the active peptide revealed five additional peptides, annotated as unknown proteins. Aligning all eight PSY homologs revealed high similarity in the domain corresponding to the active PSY1 domain, whereas the entire precursor peptides were much less similar (Table 2 and Figure 2). This indicates that the mature peptides show functional similarity, while processing of the precursor peptides may diverge between the homologs. All eight PSY precursor peptides were predicted to contain a signal peptide for secretion in their N-terminal end, a crucial feature of secreted signaling peptides (Figure 2). 

The site for tyrosine sulfation (DY) is conserved across all eight homologs, while the two prolines found to be hydroxylated and glycosylated in PSY1 are not completely conserved in PSY2-8. However, a single proline is found in the same position or further upstream in five of the seven isoforms (Figure 2). The complete conservation of the Asp-Tyr motif emphasizes its importance for PSY activity. Tyrosine sulfation is essential for affinity as well as activity for the tyrosine sulfated peptides PSK and RGF where PSK lacking one of two sulfations at its tyrosine residues has 10 times lower affinity for its receptor [8,9] and un-sulfated RGF1 has 185-fold reduced affinity for one of its three receptors, RGF receptor1 (RGFR1) [16,43]. Desulfated PSY1 only very weakly induced H^+^ pumping, demonstrating the importance of tyrosine sulfation for full activity, but H^+^ pumping is independent of both hydroxylation and glycosylation [30]. Glycosylation, however, was shown to be highly important for the post-translationally modified peptides of the CLAVATA3 (CLV3)/EMBRYO SURROUNDING REGION (CLE) family where post-translational arabinosylation of CLV3 and CLE-RS peptides proved to be critical for both biological activity and affinity to their receptors [44,45]. In both cases hydroxyproline serves as a bridge for the critical glycosylation, giving this modification an important role as well. Furthermore, proline hydroxylation was recently shown to have a high impact on proteolytic cleavage of CLE40, though not on its interaction with its receptor [46].

**Figure 3:**
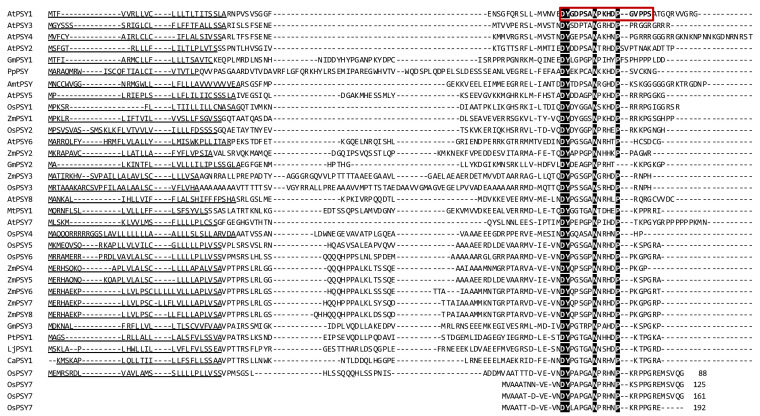
Alignment of a selection of PSY homologs in different plant species and mosses based on translated complementary DNA sequences. Amino acid sequences for the PSY homologs are from *Arabidopsis thaliana* (AtPSY); *Amborella trichopoda* (AmtPSY); rice, *Oryza sativa* (OsPSY); maize, *Zea mays* (ZmPSY); soybean, *Glycine max* (GmPSY); chickpea, *Cicer arietinum* (CaPSY); *Lotus japonica* (LjPSY); *Populus trichocarpa* (PtPSY); *Medicago truncatula* (MtPSY) and *Physcomitrella patens* (PpPSY). OsPSY7 which includes four copies of the mature peptide is found at the bottom of the alignment arranged to highlight the repeated sequence. The positions in the sequence are included. The red box and letters in bold indicate mature PSY1. Black boxes indicate conserved amino acid residue. Underlined amino acids are predicted secretion signal peptides.

Searching for PSY homologs in other plant species revealed that PSY signaling is common in plants (Figure 3) but is not found in other organisms. Homologs to *Arabidopsis* PSYs were found in many plant species, belonging to mainly monocots and dicots, but also a single homolog was found in the moss *Physcomitrella patens* (Figure 3). Finally, the RaxX proteins from *Xanthomonas* were found. Like the *Arabidopsis* PSY homologs, the prepropeptide sequences of different plant species show high variability while the PSY domain is well conserved. 31 of the 32 aligned sequences have all five conserved amino acids from the active PSY domain; DY, N, H, and P. Only one sequence from soybean (*Glycine max*; GmPSY2) lacks the complete conserved domain as it carries four out of the five amino acids, but is lacking the last P (Figure 3). The fact that the PSY domain is so well-conserved across multiple plant species, emphasizes the proposed important role of the peptide family in plant development. 

### 4.2. SBT–A Possible Modifier of PSYs

Post-translationally modified peptides require proteolytic processing to release the biologically active, mature peptide. (Figure 1) IDA (Inflorescence Deficient in Abscission) peptides are processed by subtilisin-like serine proteases (SBTs), and plants lacking SBT activity phenocopied the abscission defect of ida mutants, a phenotype which was restored when treated with active IDA peptide [47]. This indicates that insufficient processing may be the limiting factor for peptide signaling. Identifying enzymes involved in processing of peptide precursors is of great interest, but identification of putative processing proteases is challenging due to the large number of candidates and scarce information on conserved motif sites. In yeast and animal systems, enzymes cleave precursor peptides at pairs of basic amino acids, a motif which is not present in plant precursor peptides [4,48].

One can speculate that enzymes involved in processing of other tyrosine sulfated peptides also function in PSY processing or have a homolog which does. The protease family of SBTs seems to be promising candidates as these are involved in processing of both PSKs and RGFs. SBT1.1 is required for processing of PSK4 where it cleaves three residues upstream of the N-terminus of the mature peptide at the sequence motif RRSLVL/HTDY. Despite the recognition motif being well conserved in five of the six PSK homologs, SBT1.1 cleavage is very specific towards PSK4 [27]. The SBT1.1 recognition is not present in any of the PSY precursor peptides (Table 3). Less substrate specific proteases, SBT6.1 and SBT6.2, are found to be involved in RGF6 processing [28]. SBT6.2 is categorized as a non-specific enzyme involved in protein turnover and may be involved in processing of several peptides [49]. SBT6.1 activity was found to be essential for RGF6 activity, where SBT6.1 cleaves RGF6 at two sites; RRLR and RRAL (RXLX; RXXL). The same SBT is involved in processing of leucine zipper transcription factors (bZIPs) [50,51], pectin methyltransferases (PMEs) [52], and rapid alkalinization factor (RALFs) [53], proposing a broader substrate specificity. Despite SBT6.1 cleavage being required for RGF6 activity, SBT6.1 processing does not yield the mature, active peptide [28]. Recently it was suggested that SBT6.1 acts in the early Golgi compartment to ensure continued passage for RGF6 along the secretory pathway and hence additional posttranslational modifications and processing in post-Golgi compartments [26]. The recognition site for SBT6.1 is found in PSY1-5, indicating that PSY peptides may be processed in a similar way (Table 3).

In *Arabidopsis* the SBT family comprises 56 genes grouped in six subfamilies [54]. Out of these 56 SBTs, a single one encodes a phytaspase, possessing extreme aspartate specificity [55]. This aspartate-dependent protease, SBT3.8, has recently been shown to be involved in the biogenesis of both PSK and RGF peptide, yielding the active peptides, cleaved at their N-terminal site [26,56]. Tyrosine-sulfated and non-sulfated RGF6s were cleaved by SBT3.8 at similar rates, indicating that sulfation of the tyrosine upstream of the aspartate motif does not affect processing activity [26]. Mature peptide sequences of PSY1-8 all carry the same DY-motif at their N-terminus as RGF6 and PSK1, which are found to be processed by SBT3.8, which suggests that SBT3.8 may be involved in PSY processing as well (Table 3). 

The known steps in processing of RGF6 emphasize the complexity of peptide processing. Cleavage by SBT6.1 is required for RGF6 activity; however, the active peptide is not produced without processing at its N-terminal end. SBT3.8 is able to cleave at the DY-motif, producing a fully processed RGF6 peptide at the N-terminal end, although *sbt3.8* mutants did not copy the phenotype of *rgf6* mutants. This indicates that other proteases act redundantly with SBT3.8 [26]. RGF6 processing occurs in several compartments from cleavage of the secretion signal in the ER, processing by SBT6.1 in cis-Golgi to additional processing and posttranslational modification further downstream in the secretory pathway [26]. 

Successful processing of CLE40 of the CLE-family of post-translationally modified peptides was shown to be dependent on hydroxylated proline [46]. The proline hydroxylation was proposed to protect the precursor peptide from SBT-mediated processing of an internal cleavage site [46], and was thus not directly affecting the bioactivity of CLE40 but instead reassuring correct peptide processing. A similar mechanism may give rise to mature PSY1 which is the only peptide of the PSY family known to carry hydroxylated proline as a posttranslational modification (Figure 2) [5]. Such peptide protease protection may be of high importance to ensure correct C-terminal processing. No proteases have yet been identified as part of the C-terminal processing of tyrosine sulfated peptides, and searching for cleavage motifs is highly challenging as the full length of most of the mature peptides is not known.

So far, the family of SBTs, and SBT6.1 and SBT3.8 in particular comprises the most promising processing enzymes to be involved in PSY processing, Of the 56 SBTs existing in *Arabidopsis,* many are still not characterized, and their substrates are not yet identified [49]. This raises the possibility that several SBTs are involved in cleavage and fine trimming of PSY precursor peptides to reveal the mature peptide. 

### 4.3. The PSY Peptide Family Serves Different Roles in Plant Growth and Development

**Figure 4:**
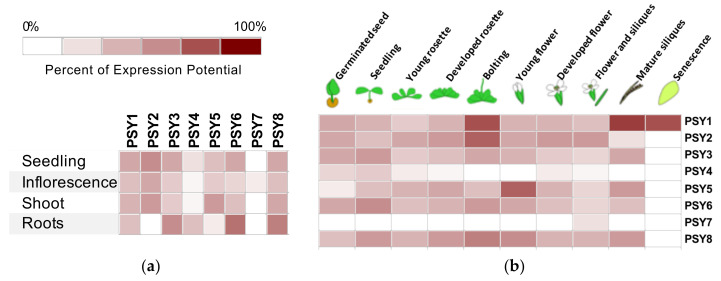
Transcript levels of PSY1-8 based on mRNAseq data from Genevestigator [41]. PSY expression in (**a**) different anatomical parts of the plant and (**b**) at different developmental stages. Expression is represented as percent of expression potential for each gene and presented on a log2 scale. 100% corresponds to maximal expression of a single gene. Only data for wildtype genotype and non-treated plants are included.

To provide an outline of the physiological role of the PSY peptide family, the expression pattern of each of the eight PSYs was investigated. An in silico analysis of PSY1-8 gene expression was conducted using Genevestigator [41]. All eight PSYs are included in the mRNAseq data while only PSY1-3 are included in microarray studies as these three genes are part of the standard ATH1 microarray chip for *Arabidopsis.* The expression data available on tissue distribution and plant development from mRNAseq studies are presented in Figure 4. 

In general, PSY1 was the peptide with the most widespread expression pattern, being nearly equally expressed in all tissues (Figure 4a) and when comparing to the other family members PSY1 is quite highly expressed through all developmental stages, although the highest expression was seen in later plant development during bolting, late silique development and senescence (Figure 4b). The same expression pattern was observed for PSY8, though root tissues had higher PSY8 expression compared to other tissues (Figure 4). PSY2 was only expressed in green parts of the plant and showed the highest expression during rosette development and bolting, a pattern similar to that of PSY5 with low expression in the root, although PSY5, unlike PSY2, was seemingly also involved in flower development. PSY3 and PSY6 both had a wide expression profile with highest expression levels in roots and relatively high expression during all developmental stages (Figure 4). Two of the 8 PSYs, PSY4 and PSY7, showed a more distinct expression pattern; PSY4 was expressed mainly in roots and at early developmental stages and PSY7 expression was limited to flower tissues during late flower development (Figure 4).

**Figure 5:**
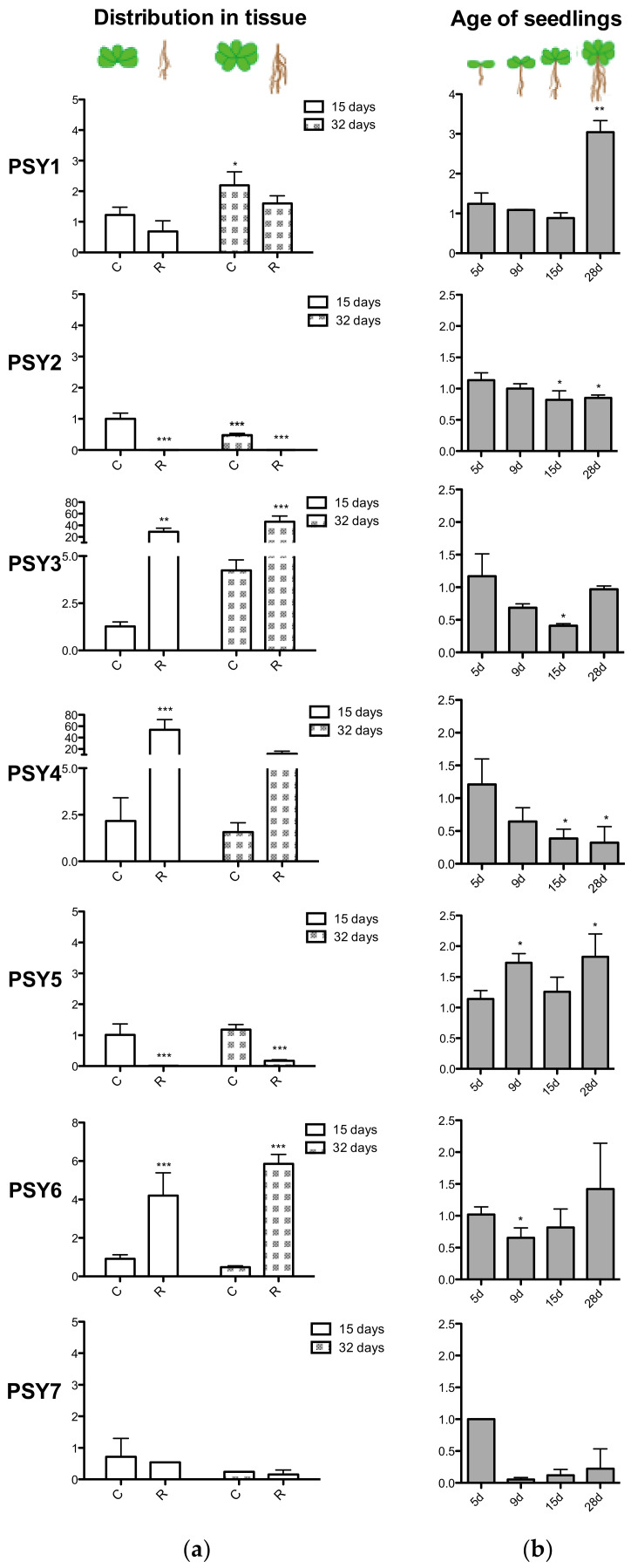
qPCR analysis of PSY1-7. (**a**) 15- and 32-days-old seedlings divided into shoot (C) and roots (R) before RNA collection. (**b**) RNA collection of entire seedlings after 5, 9, 15 and 28 days. Transcript levels were normalized to actin and relative to (**a**) the 15d cotyledon sample and (**b**) the 5d sample. Note that the y-axis units differ. Statistical analysis was done with One-way ANOVA, Dunett’s post-test with (**a**) the 15d shoot sample and (**b**) the 5d sample as reference. PSY7 expression was nearly not detectable and a transcript level of 1 is not comparable. * indicates *p* < 0.05, ** indicates *p* < 0.01, *** indicates *p* < 0.001. PSY8 was identified at a late stage and was therefore not included in the qPCR analysis.

To verify the tendencies found in the database search, qRT-PCR was performed on RNA isolated from wildtype *Arabidopsis* seedlings. The transcript level of PSY1 increases with age and was otherwise widely expressed in both green parts and roots (Figure 5). In good agreement with Genevestigator, the qPCR analysis showed that PSY2 and PSY5 were almost exclusively expressed in aerial parts of the plant (Figure 5a), with PSY2 transcript level decreasing with age (Figure 5b). PSY3, PSY4, and PSY6 were mainly expressed in root tissues, with transcript levels for PSY3 and 4 highly exceeding levels of the other homologs, which is in slight disagreement with expression data from Genevestigator (Figure 4 and Figure 5a). Furthermore, expression of PSY4 significantly decreased with age. Transcript levels of PSY7 were nearly not detectable and may correlate with PSY7 only being expressed in flower tissues which was not part of the qPCR analysis. A transcript level of 1 is thus not representable for PSY7 expression. PSY8 was identified at a late developmental stage and was therefore not included in the qPCR analysis.

Because the spread of secreted signaling peptides in the apoplastic cell wall space is restricted [57], it is a reasonable assumption that the peptides have a physiological role in the area close to their origin of expression. However, it is not certain that the level of PSY transcript corresponds to the level of mature and active peptide, as the activity is dependent on posttranslational modifications and peptide processing. Thus, expression data from Genevestigator and qPCR experiments provide only indications of the physiological role of PSY1-8. The expression patterns of the PSY homologs are quite distinct and should be investigated further. PSY4 and in particular PSY7 show tissue specific expression patterns which may assign them specialized roles in plant growth and development. Furthermore, searching the Genevestigator database based on the microarray ATH1 dataset, showed that PSY2 had very high transcript levels in guard cell protoplast. Both tissue- and cell-specific expression patterns should be examined further to find the highly specific physiological roles for more of the PSY homologs.

### 4.4. Involvement of the PSY Homologs in Plant Stress Responses

**Figure 6:**
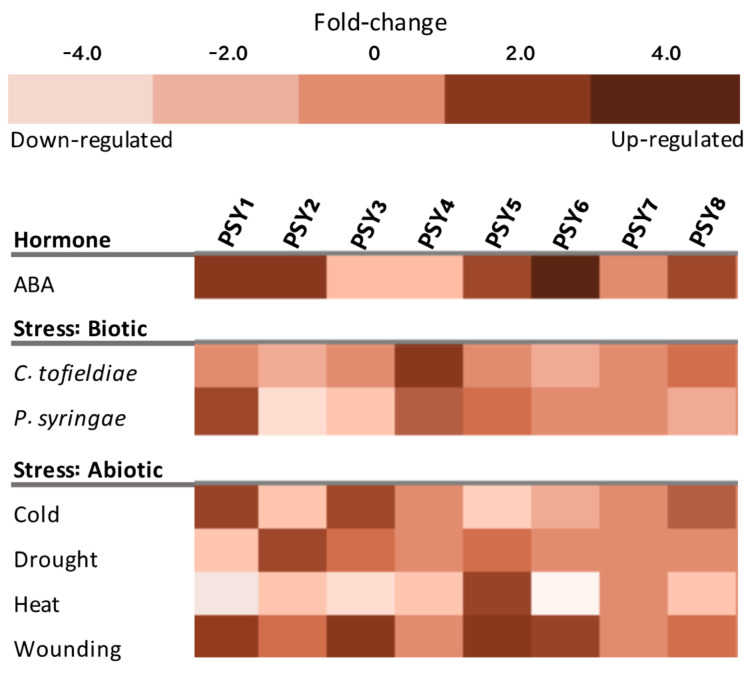
Transcript levels of PSY1-8 affected by selected perturbations based on mRNAseq data from Genevestigator [41]. ABA treatment corresponds to 6 h incubation in 50 µM ABA solution compared to no treatment. *C. tofieldae* response was measured 6dpi in roots, *P. syringae* response was measured 1hpi in rosette leaves and compared to untreated roots and leaves, respectively. Cold stress is 4 °C for 3 h compared to 1 h. Drought stress refers to mild drought stress with soil water content of 1.2 g water g^−1^ dry soil for 6d compared to no stress. Heat stress refers to 3-week-old rosette samples grown at 21 °C, 16 h light regime, exposed to 37 °C for 6 h compared to rosette samples receiving no heat stress. Wounding refers to wounded leaves measured 3 h after wounding compared to 1 h. Effect of selected treatments is illustrated as fold-change.

The in silico analysis of PSY1-8 gene expression was additionally extended to investigate whether the PSY homologs are involved in plant responses to different stresses. Seven of the eight PSYs showed substantial changes in transcript levels upon various treatments, indicating that they are regulated at the transcriptional level (Figure 6). Only PSY7 was not affected by any perturbations. PSY1, PSY2, PSY5, PSY8 and in particular PSY6 were upregulated in response to abscisic acid (ABA). PSY3 and PSY4 were downregulated. As part of an immune response to pathogens, PSY1 was previously shown to be part of a mechanism downregulating the salicylic acid response, which unregulated would have left the plant more vulnerable [34]. The PSY homologs may function in a similar mechanism to regulate ABA signaling. ABA is involved in a variety of environmental stress responses, including both biotic and abiotic stresses [58]. Particularly abiotic stress responses activate the ABA signaling pathway, though a correlation between PSYs being upregulated by both ABA and abiotic stresses is not observed in Figure 6. In general, the seven PSYs that are affected by the different perturbations, have very diverging responses to the tested abiotic stresses (Figure 6). These very differential responses to external factors indicate that the different PSYs are involved as signaling molecules for different conditions in the plant. 

PSY1 and PSY4 had the most distinguishable responses to biotic stresses, PSY4 being upregulated both in response to *C. tofieldiae* and *P. syringae*, while PSY1 only responded to *P. syringae* where expression was greatly increased (Figure 6). *C. tofieldiae* is a non-pathogenic fungus, an endophyte beneficial for root growth under phosphate-deficient conditions [37]. PSY4 was highly expressed in root tissues (Figure 5), indicating that PSY4 and *C. tofieldiae* may function in collaboration to ensure proper root growth under scarce conditions. In agreement with previous findings of *P. syringae,* a pathogenic Gram-negative bacteria, inducing PSY1 expression upon plant infection, PSY1 was upregulated in response to this pathogen (Figure 6) [34]. PSY4 expression was likewise induced by *P. syringae* infection, indicating that PSY4 may be involved in a similar mechanism. 

### 4.5. The PSY Receptor(s) 

**Figure 7:**
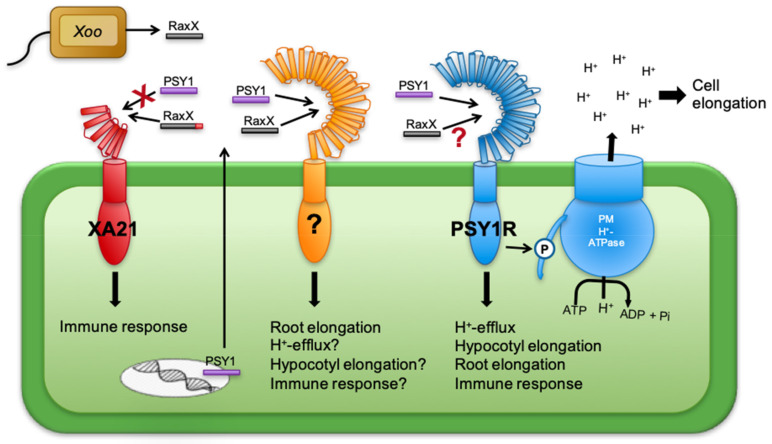
Model of PSY1 and RaxX signaling pathways. RaxX, a pathogenic molecule closely mimicking PSY1, is recognized by the immune receptor XA21, but only when carrying its C-terminal domain. XA21 does not bind PSY1. The PSY1 signaling pathway involves the PSY1 receptor (PSY1R) which induces hypocotyl and root elongation, immune response and H^+^-efflux by activating the plasma membrane (PM) H^+^-ATPase. PSY1 and RaxX induce root elongation using a signaling pathway not including PSY1R, suggesting an additional receptor recognizing the conserved PSY domain present in all PSY homologs as well as RaxX.

In order to successfully infect plants, pathogens have found clever ways to gain evolutionary advantages. The bacterial pathogen *Xoo* produces a molecule, RaxX, which greatly mimics the active peptide sequence of the PSY homologs [33]. Pruitt et al. (2017) found that RaxX also mimics the growth promoting activities of PSY peptides by promoting root growth. Treating *psy1r* mutants with RaxX or PSY1 increased root growth in a similar manner. These findings indicate that PSY1R is not the receptor for RaxX and suggests that PSY1 has another, unidentified receptor functioning in promoting root growth. This putative receptor is hypothesized to require its substrates to have the amino acid sequence conserved between the PSY1 and the RaxX pathogenic molecule, thus also being a possible receptor for the rest of the PSY peptide family (Figure 2 and Figure 7). Here PSY3, PSY4, and PSY6 seemingly functioning in root growth, are likely also to be ligands for this receptor. Furthermore, it was found that the rice XA21 immune receptor was able to recognize RaxX but not PSY1 [33,35]. RaxX could only interact with XA21 when carrying its C-terminal domain of three amino acids, PPR, though binding was independent of its five-amino-acid N-terminal domain, emphasizing the high specificity of the receptors for their peptide ligands [33]. The model presented in Figure 7 shows the receptor for PSY1, PSY1R, known to phosphorylate and thus activate the PM H^+^-ATPase, thereby promoting elongation of cells. PSY1 is so far the only ligand identified for PSY1R, however, no binding studies have been made and it is not known how much of the mature peptide is needed for PSY1R perception. If the C-terminal part of PSY1 is not important for interaction with PSY1R, other PSY homologs may also be involved in this signaling pathway. The hypothesized receptor which is activated by both RaxX and PSY1, and thus possibly also other PSY1 homologs, may function in a mechanism similar to that of PSY1R in order to induce root elongation. 

## 5. Conclusions

The PSY peptide family of small post-translationally modified signaling peptides constitute eight homologs in *Arabidopsis* named PSY1-8. PSY homologs are present in several higher plant species where several members are found. In contrast, only a single homolog is found in the moss *Physcomitrella*. The PSY peptides share a highly conserved motif, containing the characteristic DY residues for tyrosine sulfation. The PSY homologs are much less similar in their propeptide sequences, indicating that they are processed differently throughout the secretory pathway. AtPSY1 is the only peptide for which the mature peptide sequence and modifications are established. Its modifications include one sulfated tyrosine and two hydroxylated prolines, one of which is further arabinosylated. Only the site for tyrosine sulfation is conserved across all eight AtPSYs and it is yet to be established whether any of the seven PSY1 isoforms is hydroxylated and possibly glycosylated. The low similarity in PSY propeptide sequences is likely to influence how they are proteolytically processed. Based on recent findings in the processing of other tyrosine sulfated peptides, SBTs, and in particular SBT3.8 and SBT6.1, seem promising candidates to be involved in liberating the mature PSY peptides. 

The physiological role of the *Arabidopsis* PSY peptides is indicated by their expression profiles based on mRNAseq data from Genevestigator and a qPCR analysis. PSY1 and PSY8 are present throughout the plant, PSY2 and PSY5 are mainly expressed in aerial parts while PSY3, PSY4, and PSY6 are mainly expressed in root tissues. PSY7 is seemingly only expressed during late flowering. This indicates that the different PSY isoforms might serve different and distinguished functions in the plant. In response to developmental cues, the peptides were either up- or downregulated, indicating that they, at least to some extent, are regulated at the transcriptional level. Interestingly, some of the PSY homologs were greatly upregulated in response to ABA and pathogenic infections, functions already established for PSY1, suggesting that more PSY peptides are involved in similar mechanisms. 

The variability in propeptide sequences and thus the proposed differential processing, distinct expression patterns, and diverging responses to external factors all give rise to many speculations about the physiological role of the PSY peptides. So far, only one receptor, PSY1R, is known to be involved in the PSY1 signaling pathway. As the binding properties to this receptor are not yet established, it is not known whether PSY2-8 are also substrates for it. Because the PSY homologs seem to serve many different and highly diverging functions, it is a reasonable assumption that more receptors, recognizing these sulfated peptides, must exist. A receptor recognizing both PSY1 and the pathogenic PSY1-like molecule, RaxX, is proposed. This receptor is presumably involved in root elongation and may function in a mechanism similar to that of PSY1R. Involving H^+^-efflux by activation of the PM H^+^-ATPase, this putative receptor is hypothesized to bind to a motif with amino acid residues representing the conserved motif between PSY1 and RaxX. It might be a receptor for the rest of the PSY peptides as they all contain the motif. Because such a receptor recognizes both RaxX and PSY1, the seven PSY homologs, PSY2-8, are also likely to be ligands for it. 

This review offers many new speculations about the family of PSY peptides. Their expanding family size and the extensive expression profiles indicate that their functions in plant growth and development are prevalent. The many unanswered questions arising here must be experimentally confirmed and further investigated. Further studies will provide links between the molecular cell responses and the physiological effects of signaling peptides. 

## Figures and Tables

**Table 1:** Primer sequences used for qPCR.

Product	Forward Primer	Reverse Primer
PSY1	5′-TTGATGGTGAACGTTGAGGACT-3′	5′-CGGTTGCTGACGGAGGAA-3′
PSY2	5′-TCCACTTGTAACATCTTCATCC-3′	5′-ACTTGTAGTCCCTGTCTTCACA-3′
PSY3	5′-TCTCTCTTCTGCTCGCATCA-3′	5’-TGCTCACCATCAATGACCGT-3′
PSY4	5′-CAATCGTCTCTTCGGCTCGA-3′	5′-TGATCTACCCCTCACCATCA-3′
PSY5	5′-CTCTATCCAGCTCGACGGT-3′	5′-CTTCACACCCACCTCCTCAC-3′
PSY6	5′-ACCAAGGGACAGGAATTGA-3′	5′-CAACGGTCATCATCCTCCTT-3′
PSY7	5′-GAACCAGGCCCTAACCCAAT-3′	5′-TCATCTTTGGTGGAGGTGGC-3′
Actin-2	5′-CTTGCACCAAGCAGCATGAA-3′	5′-CCGATCCAGACACTGTACTTCCTT-3′

**Table 2:** PSY homolog’s similarity to PSY1.

Gene	Precursor Peptide	PSY Domain
PSY1 (At5g58650)		
PSY2 (At3g47295)	57.3%	84.6%
PSY3 (At2g29995)	44.7%	84.6%
PSY4 (At1g07175)	43.9%	92.3%
PSY5 (At5g53486)	31.1%	69.2%
PSY6 (At1g74458)	32.7%	53.8%
PSY7 (At3g49305)	34.8%	69.2%
PSY8 (At3g47510)	26.6%	38.1%

**Table 3:** Enzymes involved in processing of tyrosine sulfated peptides. Abbreviations: subtilisin-like serine protease (SBT), phytosulfokine (PSK), root meristem growth factor (RGF), plant peptide containing tyrosine sulfation (PSY).

Modifying Enzyme	Target Peptide	PSY with Motif	Reference
SBT1.1	PSK		Srivasta et al., 2008 [27]
SBT3.8	PSK	PSY1-8	Stürhwoldt et al., 2020 [56]
SBT6.1	RGF	PSY1-5	Ghorbani et al., 2016 [28]
SBT6.2	RGF		Ghorbani et al., 2016 [28]
SBT3.8	RGF	PSY1-8	Stürhwoldt et al., 2020 [26]
